# Time’s up: mutation rate and lifespan

**DOI:** 10.1038/s41392-022-01122-8

**Published:** 2022-08-12

**Authors:** Anjali Cremer, Wesley T. Abplanalp, Michael A. Rieger

**Affiliations:** 1 Department of Medicine, Hematology/Oncology, Goethe University Frankfurt, Frankfurt am Main, Germany; 2 Frankfurt Cancer Institute, Frankfurt am Main, Germany; 3 German Cancer Consortium (DKTK) and German Cancer Research Center (DKTK), Heidelberg, Germany; 4 Institute for Cardiovascular Regeneration, Centre of Molecular Medicine, Goethe University Frankfurt, Frankfurt am Main, Germany; 5 German Center for Cardiovascular Research DZHK, Partner site Frankfurt Rhine-Main, Berlin, Germany; 6 Cardiopulmonary Institute, Goethe University Frankfurt, Frankfurt, Germany; 7 EnABLE Consortium, Goethe University Frankfurt, Frankfurt, Germany

**Keywords:** Cancer, Molecular medicine

In a recent study published in *Nature*, Alex Cagan, Adrian Baez-Ortega and colleagues found that many mammalian species present a similar somatic mutational load at the end of life. The mutational burden is independent of lifetime and body mass, only varying by a relatively small fold change among species.^1^ These data may suggest that mutational burden predicts mortality.

Already 70 years ago researchers hypothesized that cancer and aging were caused by an accumulation of mutations in somatic cells.^2^ While somatic mutations in recurrent cancer-driver genes clearly promote carcinogenesis, studying the influence of somatic mutations and tissue mosaicism for aging and age-associated diseases remained challenging, due to the enormous heterogeneity of small mutated clones. With the recent advancement in DNA sequencing technologies, long-standing questions regarding the evolution of somatic mutations can now be addressed.

It was the epidemiologist Richard Peto who found an incongruity between logic and biological reality, known as Peto’s paradox. He noted that if somatic mutations develop at a linear rate, and mutations predict cancer, then large animals which have more cells and hence could acquire more mutations, should die sooner than smaller animals. However, this is not the case.^3^

**Fig. 1:**
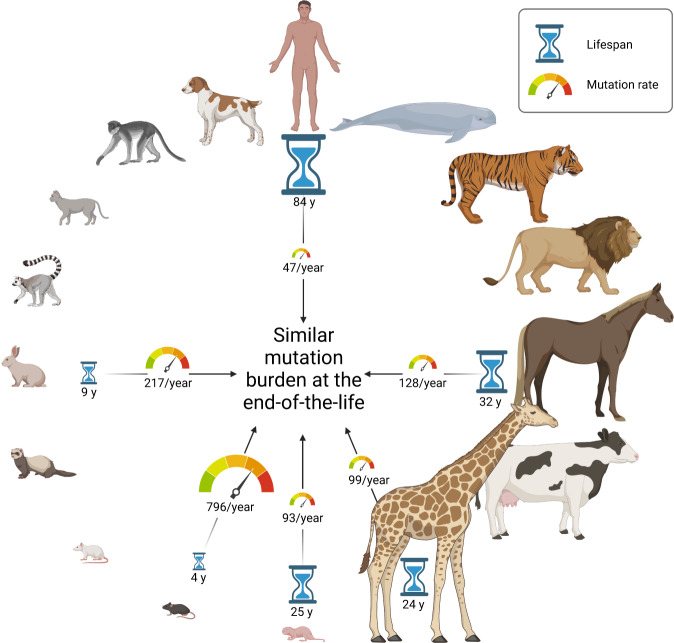
Mutation rate is inversely correlated with lifespan. Cagan et al.^1^ demonstrate by comparative whole-genome sequencing of clonal colon crypts that the mutational rate of somatic mutations (tachometer) is inversely correlated with the lifespan (hourglass) in mammals across 16 different species. However, the mutation burden at the end of life is highly similar among these species. This observation is largely independent from the respective body mass. The figure was generated with BioRender

Cagan et al. provided experimental data partly explaining Peto’s paradox—only made possible through today’s technology.^1^ The authors compared whole-genome sequencing analyses from micro-dissected single colon crypts of 16 mammalian species. The tissue is appropriate to study naturally occurring somatic mutation rates, given its conserved anatomical status and clonal origin. Indeed, the authors verified that mutation accumulation is linearly increasing over time within a species, as previously demonstrated in humans.^4^ However, the relative mutation rate is species-dependent and highly variable among species. Mutations presented in three consistent COSMIC mutation signatures across species (equivalent to human SBS1, 5 and 18). Importantly, the authors demonstrated that the somatic mutation rate is inversely proportional to lifespan in 16 mammalian species (Fig. 1). This is illustrated by comparing the high mutational rate per year of the short-lived mouse (~3 years) versus the slow rate of the long-lived naked mole rat (>25 years). The other striking aspect of the study is that species as diverse as mice, giraffes and humans, all with different respective lifespans and body sizes, present a similar number of somatic mutations at the end of life. The somatic mutation load differs only by a factor of about three-fold, while body mass shows a 39,000-fold difference among species in this study. Effectively, when the critical abundance of mutations develops, the parking meter of life will read “time expired”. The slow mutation rate in long-lived mammals indicates that controlling somatic mutation development limits cancer risk. This also explains why mammals have similar lifetime cancer incidence, as several driver mutations are required to develop a malignant transformation, despite the large difference in body mass and lifetime. However, additional cell intrinsic and environmental factors shape cancer development, which are not discussed here.

The authors report that the strong anticorrelation of mutation rate and lifespan is maintained for the three types of mutation signatures and for mutation patterns in coding regions as well as in mitochondrial DNA, suggesting that no single biological process (DNA repair pathways may differ between species) is controlling this phenomenon. Thus, total mutational load better correlates with lifespan than individual mutational processes.

Still puzzling are the causes of cellular and organismal aging. This study demonstrates a similar mutational burden at the end of life for mammals. Are the acquired mutations responsible for aging and dysfunction in cells, organs, and the whole organism? Then the mutation rate would be the pacemaker of the clock of life, and therefore a mutation threshold would limit lifespan. However, observations of individuals with inherited disabilities in certain DNA repair mechanisms resulting in a much higher somatic mutation rate do not show overt signs of premature aging.^5^ Alternatively, a certain mutation burden may be tolerated without empirically detected physiological impacts on negative selective pressure, and perhaps mutation rate evolved alongside lifespan. Animals with shorter lifetimes can tolerate higher mutation rates, so less emphasis on extensive repair mechanisms may be paid for keeping the mutation rate low.

We still know little about the potential of acquired somatic mutations to cause (rather than associate with) age-related diseases other than cancer. While a direct consequence of the accumulation of mutations on cell function and survival in terms of aging processes appears rather unlikely, the cellular interplay involved in the body’s response to impaired organ performance may be influenced. Immune cell dysfunction caused by the somatic mutations in blood cancer-related genes in blood stem cells, known as clonal hematopoiesis, has gained medical interest. While these mutations provide a competitive advantage in the stem/progenitor cell compartment, they also cause detrimental functional changes in immune cells that have negative effects on the progression of cardiovascular diseases and thereby impact human aging in an organismal interplay. Aging is a multifactorial and complex process and is not solely dominated by the accumulation of somatic mutations in individual cells. Rates of telomere shortening, protein aggregation, and epigenetic alterations may equally predict mortality. Somatic mutations in certain cell types in multicellular organisms may impact on the stability of the network and cause unpredictable and disruptive forces on the organism.

Many follow-up questions come to light when considering the study by Cagan et al. What are the molecular processes that determine and control mutation rates in divergent species? What are the selective pressures driving longer lifespans? Does the accumulation of somatic mutations in all tissues follow the same trends for a given species? This manuscript supports the discussions as to whether the mouse is a suitable model organism. For the data in this investigation, mice fit the model of aging and disease very well, however, the control of DNA integrity might differ between humans and mice.

The study by Cagan et al. is a milestone study that can provide a new framework for how to view not only human health and disease, but mammalian life as a whole.
